# The pre-exposure prophylaxis (PrEP) consciousness of black college women and the perceived hesitancy of public health institutions to curtail HIV in black women

**DOI:** 10.1186/s12889-020-09248-6

**Published:** 2020-07-28

**Authors:** Rasheeta Chandler, Shawnika Hull, Henry Ross, Dominique Guillaume, Sudeshna Paul, Nikita Dera, Natalie Hernandez

**Affiliations:** 1 Nell Hodgson Woodruff School of Nursing (NHWSON), Emory University, 1520 Clifton Rd., NE, Atlanta, GA 30322-4027 USA; 2 George Washington University, Prevention and Community Health, 1918 F Street NW, Washington, D.C, 20052 USA; 3 University of Rochester, Center for Community Practice, 601 Elmwood Ave, Rochester, NY 14627 USA; 4 Morehouse School of Medicine, Community Health and Preventive Medicine, 720 Westview Drive, Atlanta, GA 30310 USA

**Keywords:** PrEP (pre-exposure prophylaxis), Black women; online education, HIV, College students, Prevention

## Abstract

**Background:**

Consistent use of Pre-Exposure Prophylaxis (PrEP), a biomedical intervention for HIV seronegative persons, has been shown to significantly decrease HIV acquisition. Black women are a viable population segment to consider for PrEP use as their HIV incidence is overwhelmingly higher than all other women groups.

**Methods:**

We developed and piloted a cultural- and age- appropriate PrEP education intervention to determine Black college women’s: 1) perceptions of and receptivity to PrEP use; and 2) preferences for PrEP information delivery.

**Results:**

We recruited *N* = 43 Black college women. Most of our sample were sophomore and Juniors of whom identified as heterosexual (83%) and single (67%). Over 50% of young women had never been HIV tested and only 28% had been tested in the last 6 months; however, 100% of the women believed their HIV status was negative. Prior to participating in the study, most Black college women (67%) had not heard about PrEP and were unsure or apprehensive (72%) to initiate PrEP. The Black college women indicated that our educational intervention was extremely helpful (67%) for understanding and learning about PrEP. Post participating in our PrEP education module, regardless of delivery modality, participants reported being likely (62.55–70%) to initiate PrEP in the future.

**Conclusions:**

Results indicate that Black college women would strongly consider PrEP when provided with basic knowledge, regardless of delivery modality. Participants also showed greater appreciation for in-person delivery and found it to be significantly more helpful and of greater quality for learning about PrEP; comprehension or perceived usefulness of PrEP-related content was relatively the same between groups. PrEP content delivery -- via in-person or online methods – is contingent on learning style and presentation.

**Trial registration:**

This study has been registered under the ISRCTN Registry as of July 6, 2020. The trial registration number is ISRCTN14792715. This study was retrospectively registered.

## Background

Pre-exposure prophylaxis (PrEP) is currently available in the United States as an FDA-approved measure that employs a daily oral dose of emtricitabine/tenofovir disoproxil fumarate (Truvada) [1, 2]. Populations at high risk for HIV acquisition may especially benefit from the prophylactic use of ART treatment as prevention in combination with other prevention methods (e.g. condoms) [3, 4]. When used consistently, PrEP, the provision of antivirals to non-HIV infected persons, has been shown to significantly decrease the risk of acquiring HIV [3–7]. Although approved by the FDA in 2012, the application of PrEP guidelines has been inconsistent particularly in the context of PrEP among Black women who have demonstrated significantly low PrEP uptake despite being at high risk of HIV [8, 9]. In addition to the inconsistent application of PrEP guidelines, there has been insufficient policy infrastructure to govern access and cost concerns [10].

While PrEP is a viable addition to HIV prevention, there are concerns that PrEP as a prevention method may undermine education efforts and advocacy for condoms and other safer sexual practices [11]. Additional concerns associated with PrEP distribution are: (a) concerns of potential toxicity with long-term use (e.g. decreased bone mineral density among women who have a higher baseline risk for osteoporosis) [12, 13]; (b) direct costs associated with sustaining PrEP adherence, recommended pre-PrEP counseling services, prescribed intervals of HIV testing, and HIV surveillance [14]; (c) limited availability of PrEP in communities with greatest need (PrEP-to-need ratio; PnR) [15]; and (d) disparities in PrEP prescribing and marketing [3]. There are ethical considerations concerning the possibility that costs and access to PrEP will widen racial, ethnic, and geographic disparities [14, 16]. This may especially affect Black women, the population with the highest rate of new sexually transmitted infection (STI) acquisition/HIV infections among women and who remain at risk of being infected while attending college [12–15, 17].

### Focus populations and PrEP as HIV prevention

PrEP use has primarily been associated with men who have sex with men (MSM) [3]; however, the CDC (2018) recommends PrEP use by any “*individuals at very high risk for sexual exposure to HIV*” and acknowledges the need for more “*comprehensive U.S. Public Health Service guidelines on the use of PrEP for the prevention of sexually-acquired HIV infection*.” [17] In MSM populations, PrEP efficacy has been shown when used in concert with other behavioral interventions [3, 18].

The Henry J. Kaiser Family Foundation [19] reports that young women, including those of reproductive age, are significantly affected by HIV: nearly one-third of new HIV infections (29%) among women occur in those aged 25–34, and 14% in women aged 13–24. However, there are significant racial disparities among women and HIV rates. Black women continue to be diagnosed with HIV at disproportionately high rates relative to White and Hispanic/Latina women. In 2017, 59% of new HIV diagnoses were among heterosexual cisgender Black women. If current rates persist, approximately one in 48 Black women will be diagnosed with HIV in their lifetime [20]. This health disparity is most pronounced, and continues to expand in the Southern U.S. where Black women ages 18–24 have higher rates of sexually transmitted infection (STI)/HIV acquisition than women classified in any other racial/ethnic group [21–23]. This risk level is primarily due to higher prevalence of HIV in Black/African American communities and the fact that African Americans tend to have sex with partners of the same race/ethnicity [24]. HBCU colleges that Black women attend are often located in high HIV prevalence areas, which is a recommended guidance for PrEP use [25].

Due to disproportionate HIV rates among Black women, especially in geographic areas with a high HIV prevalence, they are important priority populations to inform about PrEP. However, limited data exist from PrEP investigations that focus on women, especially Black women living in the U. S [9, 13, 16, 26, 27]. Generally, women reported being unaware of PrEP and found the option attractive after receiving education on PrEP; and cited additional barriers to PrEP uptake including distrust of the medical system, stigma, and cost [9]. Among women who do know about PrEP, findings have shown that it is viewed as an important prevention option assuming that side effects and cost are minimal, and delivered by trusted sources [9]. No studies have been reported to date, however, on PrEP research among Black college females. The US Women and PrEP Working group (2015) have provided specific recommendations on how to introduce PrEP to women that we incorporated in this study to equip Black female college students with ways to develop and provide women with tools to seek, afford, and use PrEP effectively [13].

*Black College Women and Technology.* HIV Prevention research that is focused on college and university students continues to gain much-needed attention [28–31]. However, there is a paucity of program opportunities designed to address the unique challenges of STI/HIV transmission in Black female college students. Black female college students are heavily dependent on the availability of computerized technologies that facilitate information access, programs/activities involvement, knowledge retention, and social support through peer interaction. Incorporating technological advancements, socially relevant context, and culturally relevant persuasive strategies are important considerations for increasing Black college female HIV prevention and sexual health risk-reduction programs. Emerging research that focuses on use of various technological advancements, including interactive media (e.g. video/audio-embedded presentations, social media, gaming), for education and training suggest that computer-based interventions may meet the needs of women in this population [32].

#### Electronic-based learning (e-learning)

Novel uses of technology (e.g. computerized programs) can enhance learner-centered education via diversification of program content delivery and interactive experiences [33, 34] that are desirable and relevant. Notable advantages of this methodology include greater flexibility and access, facilitation of distance learning, improved program fidelity, and increased opportunities for collaboration. In addition to online education being an efficacious means of delivering sexual health education, it has also been shown that brief (60 min) computer-based sex education sessions, comparable to group-based multi-session interventions, promotes protective sexual behavior and knowledge attainment [35].

The purpose of this study was to establish the feasibility and acceptability of a Pre-Exposure Prophylaxis (PrEP) education curriculum targeting Black female college students attending a Historically Black College/University (HBCU), and to determine the optimal modality (online versus in-person) for delivery of the curriculum. We sought to determine if the sub-culture of young Black women who exist in the University microenvironment had any prior exposure to PrEP content (e.g. from advertisements or healthcare providers) and, if so, the extent to which the content was deemed culturally and contextually appropriate. Specifically, we conducted a between subjects randomized experiment to test the utility of an HIV prevention curriculum for Black college women that includes PrEP. We foresee the potential for this intervention to serve as a blueprint for future PrEP public health dissemination efforts that focus on unengaged high-risk groups. We implemented a community-level (defined as college campuses for this study) approach to assessing and informing perceived risk of acquiring HIV, and to enhancing PrEP knowledge, PrEP intentions, and PrEP efficacy [9].

### Theoretical framework

**Fig. 1:**
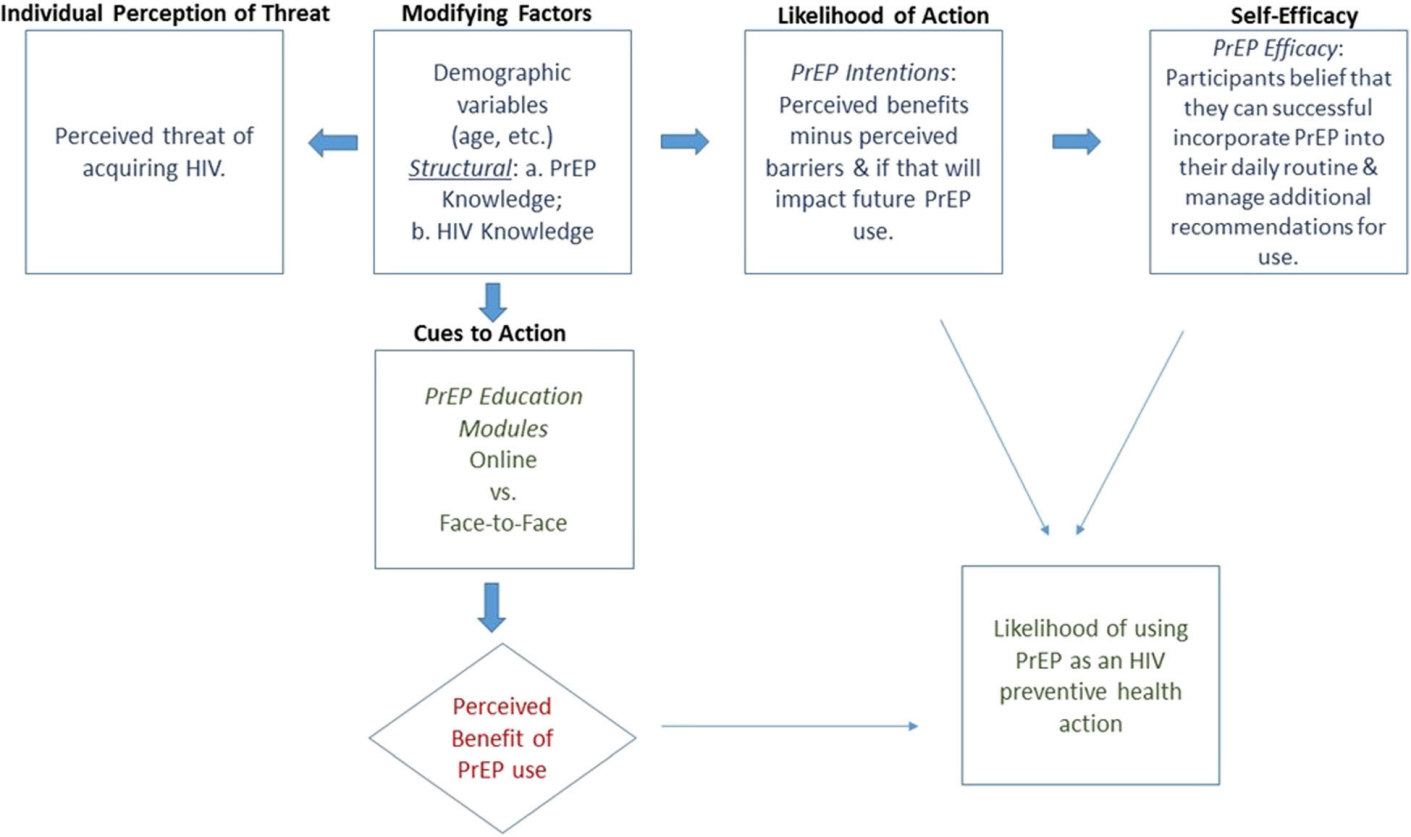
Health Belief Model Framework

Our study was guided by the Health Belief Model (HBM) [36, 37]. This framework understands that a behavior (e.g., using PrEP) is affected by several interacting factors including the perceptions of threat and benefit, cues to action, and the person’s own sense of capacity to perform the behavior. (See Fig. 1).

## Methods

### HIV prevention curriculum development

Based on preliminary research that describes preferred content and delivery modalities in HIV prevention education among Black college women, and the clinical expertise of the Principal Investigator who is a certified, practicing Family Nurse Practitioner [38, 39] we developed a one-hour PrEP Education Intervention that includes current information regarding PrEP, enhanced by interactive learning activities (e.g. games, role play), and formatted for two delivery platforms – a traditional in-person small groups and an online modular session.

### Sample and setting

#### Recruitment efforts

We replicated and enhanced our previously highly successful recruitment strategies for this population [38, 40] including campus media, word-of-mouth, and flyer distribution [hard-copy and electronic]. In all recruitment material, students were directed to the online pre-screen tool that helped determine if they met the following inclusion criteria (i.e., self-identified as Black; cis-gender Female; enrolled part-time or full-time at the designated university; between the ages of 18–24). Eligible individuals were contacted by telephone and scheduled for an initial visit which consisted of completing the consent documents, taking the pretest, and being scheduled to participate in their randomly assigned PrEP education modality [online vs. in-person]. Ineligible participants were notified via their preferred contact modality (e.g. text or email).

### Procedures

#### Approach: pilot PrEP education intervention

**Fig. 2:**
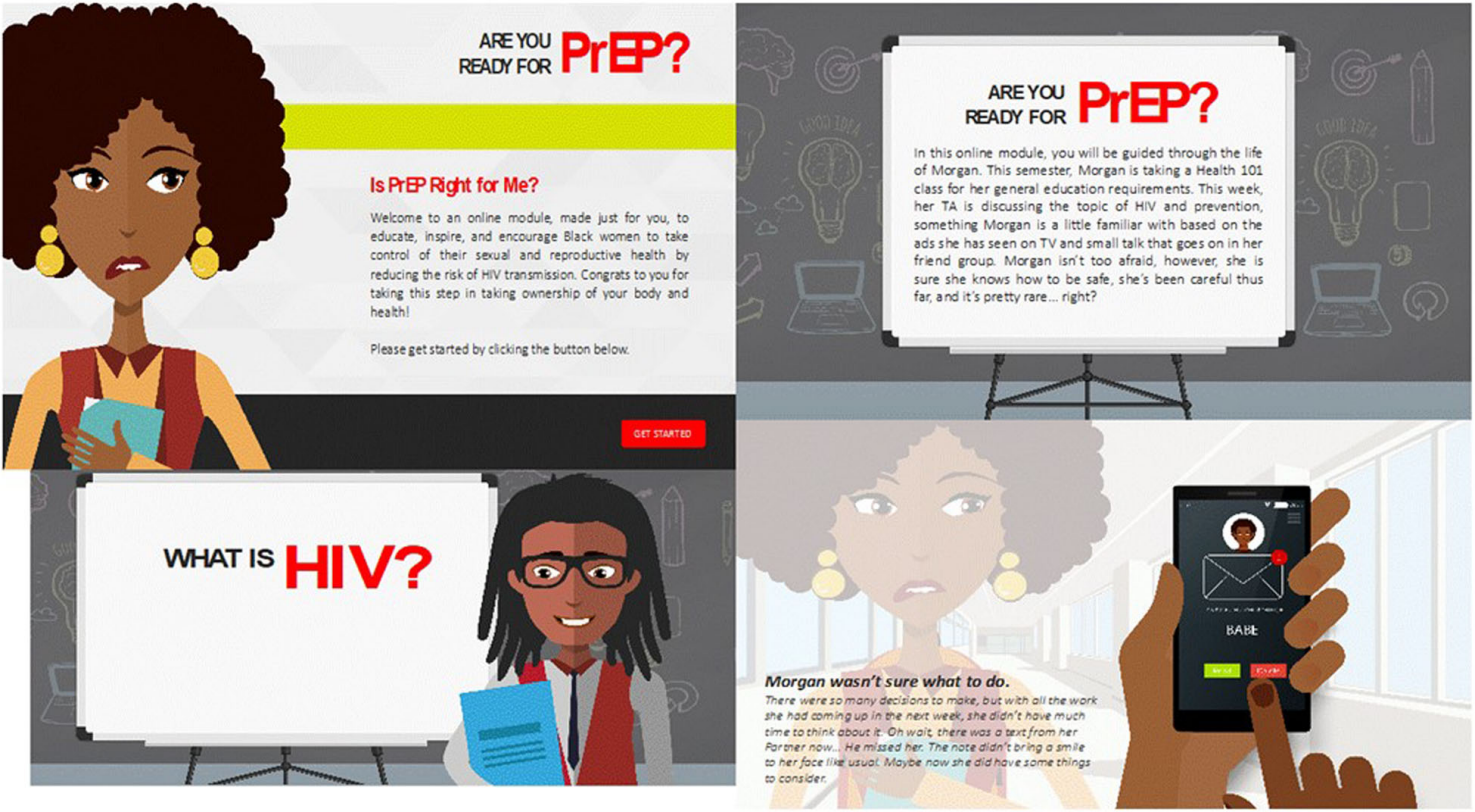
Visual Content of Online PrEP Module

Participants were assigned randomly (1,1) to a delivery platform. The in-person group sessions (*n* = 2) were facilitated by the same two members of the research team, who are both Black women and health care professionals. The online module was developed in an e-learning platform and could be accessed via an assigned username and password. Online content consisted of asynchronous audio/visual education (see Fig. 2). Table 1 outlines PrEP topics that were addressed in the single session in-person and online module. Specific details of each topic were manualized [with notes specifying adaptations for online use (i.e. to promote participant engagement, the in-person session had brief role-play segments and the online session had pop up quiz questions].

**Table 1:** PrEP Education Intervention Topical Outline

Topic (Time)	Description	HBM Theoretical Construct(s)
HIV 101 (15 min.)	Define HIV, how HIV is transmitted, and HIV impact on Black women.	Individual Perception of Risk: Risk assessment completed; Modifying Factors: HIV Knowledge; PrEP Knowledge
PrEP 101 (15 min.)	Define PrEP and risks associated with the need for PrEP use including epidemiological data supporting Black women as a high-risk group. Identify PrEP as a supplemental HIV preventive option.	
PrEP dose & implications simplified (20 min.)	Medication dose, potential side effects, other implications for use (labs, STI/HIV testing intervals); where PrEP is prescribed locally, PrEP costs, insurance coverage and drug assistance programs.	Likelihood of Action; Self-Efficacy
PrEP Empowerment (10 min.)	Empower the women to make an informed decision about their HIV prevention options, including PrEP.	Likelihood of Action; Self-Efficacy; Cues to Action [Contextually congruent in both In-person and Online platforms]: (i.e. leave condoms in your going out purse so that they are there when you need them; provided HIV information to cultivate accurate perceptions of susceptibility, scripts to initiate a conversation with healthcare provider about PrEP initiation)

#### Assessment strategies: pre-test

Eligible participants met with research staff who executed consent procedures and obtained informed consent to participate in the study. Participants were assigned a study ID number and placed in a private room to take the baseline survey using a computer device to access the digital survey. Participants were given instructions regarding the date/time/location (in-person) or uniform resource locator [url] address (online)/access code of their PrEP education session. The post-test appointment was also provided at that time. We sent reminders of all follow-up appointments via the preferred contact option selected by the student. Participants were compensated $20 at this and all subsequent visits associated with the study.

#### Post-test

We encouraged all participants to complete their scheduled post-test session (2 weeks after completing the PrEP education module), which was a re-administration of baseline surveys.

### Measures

We employed quantitative and qualitative data collection approaches to assess our expected outcomes. When this study was implemented, we did not identify studies that examined PrEP knowledge, intention, and efficacy in Black women and particularly those who are attending college. We were likewise unsuccessful in identifying validated measures that would solicit information addressing our proposed outcomes. Therefore, the pre-test and post-test questions were composed of questions generated by the research team and adapted for the focus population to ensure we adequately assessed the study outcomes. All surveys were compiled and administered via the Survey Monkey platform.

Feasibility and Acceptability questions were crafted to determine the feasibility and acceptability of recruiting and implementing a(n) in-person and online PrEP curriculum with Black College Women. Questions are noted in Table 2. We also have descriptive context detailing participants’ impressions of this PrEP curriculum and the respective delivery modality.

**Table 2:** Baseline patient characteristics

	Overall (*N* = 43)	In-person (*N* = 20)	Online (*N* = 23)	*P*-value
Age	19.95 (0.98)	19.91 (0.99)	19.99 (0.99)	0.81
Education				0.55
Freshman	11 (25.6)	5 (25.0)	6 (26.1)	
Sophomore	14 (32.6)	7 (35.0)	7 (30.4)	
Junior	14 (32.6)	6 (30.0)	8 (34.8)	
Senior	4 (9.3)	2 (10.0)	2 (8.7)	
Sex				0.48
Straight	35 (83.3)	14 (70.0)	21 (95.5)	
Gay	2 (4.80)	2 (10.0)	0 (0.0)	
Other	5 (11.9)	4 (20.0)	1 (4.5)	
Sexual behaviors				
Partnership status				0.23
Single	29 (67.4)	12 (60.0)	17 (73.9)	
Boyfriend/regular male partner	13 (30.2)	7 (35.0)	6 (26.1)	
Girlfriend/regular female partner	1 (2.3)	1 (5.0)	0 (0.0)	
In the past 12 months				
How many men you had sexual contact with				0.16
0	11 (26.2)	3 (15.0)	8 (36.4)	
1–4	25 (59.5)	15 (75.0)	10 (45.5)	
≥ 5	6 (14.3)	2 (10.0)	4 (18.2)	
How many men have you had anal sex				0.45
0	34 (81.0)	15 (75.0)	19 (86.4)	
1–4	8 (19.0)	5 (25.0)	3 (13.6)	
How many men have you had anal sex without a condom				0.07
0	34 (85.0)	13 (72.2)	21 (95.5)	
1–4	6 (15.0)	5 (27.8)	1 (4.5)	
Current HIV status				0.26
HIV negative	34 (79.1)	14 (70.0)	20 (87.0)	
Don’t know	9 (20.9)	6 (30.0)	3 (13.0)	
Recent HIV test				0.27
In the last 6 months	12 (27.9)	4 (20.0)	8 (34.8)	
6 months – 1 year ago	6 (14.0)	3(!5.0)	3 (13.0)	
1 year – 5 years ago	3 (7.0)	3 (15.0)	0 (0.0)	
Never	22 (51.2)	10 (50.0)	12 (52.2)	
Result of the last HIV test				1.00
HIV negative	21 (48.8)	10 (50.0)	11 (47.8)	
Don’t know	22 (51.2)	10 (50.0)	12 (52.2)	
Have you heard of PrEP before?				0.69
Yes	11 (25.6)	4 (20.0)	7 (30.4)	
No	29 (67.4)	14 (70.0)	15 (65.2)	
Don’t know	3 (7.0)	2 (10.0)	1 (4.3)	
How likely are you to use a PrEP pill, if available				0.51
Somewhat-very unlikely	15 (34.9)	7 (35.0)	8 (34.8)	
Not sure	16 (37.2)	9 (45.0)	7 (30.4)	
Somewhat-very likely	12 (27.9)	4 (20.0)	8 (34.8)	

PrEP Knowledge was assessed with two questions: a) Before this study, had you ever heard of PrEP; and b) What is your knowledge of PrEP? (range: where 0 is no knowledge and 10 is expert knowledge).

PrEP Intentions was assessed with one question. How likely are you to use PrEP in the future (Responses ranged from—Very unlikely [0] to Very likely [5])?

#### Focus groups

One week after participants completed their post-test evaluation, we conducted two 90-min focus group sessions. Audio-recorded sessions were conducted with 20 participants who attended either the online (*n* = 10) or in-person (n = 10) PrEP education modality, to acquire information about the feasibility and acceptability of the PrEP program. We employed a semi-structured focus group guide, which was developed by the research team, to solicit responses from the participants. An additional file that includes the focus group guide has been provided [see Additional file 1]. Sample questions included: What would be a barrier for you using PrEP? What was most helpful about the PrEP educational module? What was least helpful about the PrEP educational module? Where would be an ideal place for you to seek PrEP? Do you feel confident asking your medical provider for PrEP? What are other options that could inform you about PrEP [e.g. PrEP message alternatives/preferences]?

### Data analysis

#### Quantitative analysis

Quantitative data was analyzed using IBM SPSS Statistics (version 24), for statistical analysis. Descriptive statistics (mean/standard deviation or frequency/percent) were used to characterize the study sample, including demographics, sexual behavior and assessment of perceived risk of acquiring HIV, PrEP knowledge, PrEP intentions at baseline (pre-test). Baseline differences between the two modalities of program delivery (in-person, online) were compared using T-tests or chi-squared tests for continuous and categorical variables respectively. At post-test, the item scores corresponding to the program efficacy were evaluated to assess differences between the two groups using non-parametric Mann Whitney U tests. All statistical tests were two-sided and a *p* value of < 0.05 was considered statistically significant.

#### Qualitative analysis

Each focus group was digitally recorded and transcribed verbatim; content analysis was used to identify themes [41]. Two members of the research team independently evaluated focus group transcripts to ensure congruency with extracted themes using nVIVO software (version 12). Using iterative characterization, codes were developed [42]. Upon completion of individual analyses, the researchers convened to discuss and compare their findings, ultimately finalizing the codebook, and electing representative quotes for each theme. Trustworthiness of data was determined by: (a) debriefing after each focus group, (b) providing an audit trail of how and why study components were executed, (c) using the same basic interview guide for all groups, and (d) presentation of rich data with sample and setting descriptions [41].

## Results

The study sample consisted of 43 Black, college women attending school in Atlanta, GA. Of them, 23 (53%) and 20 (47%) were assigned to the online and in-person program respectively. Sample characteristics, including demographics, sexual behaviors, and PrEP knowledge/ intentions at pre-test are shown in Table 2. The average age of the participants was 20 years and 65% were sophomores /juniors. A majority of the sample identified themselves as heterosexuals (83%). Of all participants, 29 (67%) were single and 31 (74%) had sexual contacts with one or more men in the last year. While only 8 (19%) participants had anal sex with one or more men in the past year, 6 of those (75%) did not use a condom.

The sample majority (89%) chose not to answer questions related to their partner’s HIV status or whether they had any discussion about HIV with their partners. Of those who responded 67% had never spoken to their partners about HIV and 56% did not know about their partners HIV status. Despite their lower perceived risk of being HIV-positive, 51% of the participants were never tested for HIV and only 28% of the participants were tested/screened for the HIV virus in the last 6 months. Of those tested, 49% were confirmed HIV negative, while 51% were unsure of their test results.

Regarding PrEP knowledge and intentions, prior to participating in the study, most participants (67%) had not heard about PrEP and were unsure or apprehensive (72%) about initiating use. At baseline, there were no statistically significant differences in sample characteristics between the two modalities.

The intervention was well received by both groups. Post-test scores indicate that our educational intervention was extremely helpful for understanding and learning about PrEP (67%). In addition, more than 70% of the participants felt that the quality and usefulness of the information presented was excellent (Table 3). Compared to the online group, the in-person group provided higher scores, on the items corresponding to (a) helpfulness of the program for learning about PrEP (mean rank: 18.6 versus 25.9, *p* = .03) and, (b) the quality of information provided (mean rank: 17.22 versus 27.5, *p* = .00). Post-intervention, participants perceived their PrEP knowledge at 7 on a scale of 0 (No knowledge) to 10 (Expert knowledge). Seventy percent of the in-person participants and 62.55% of the online participants indicated that post-intervention they were somewhat to very likely to use PrEP in the future.

**Table 3:** Evaluation of post-test scores by modality of program delivery. (in-person, online)

	Overall (*N* = 43)	Modality		*P*-value*
		In-person (*N* = 20)	Online (*N* = 23)	
*How helpful was program for understanding PrEP?*				0.17
Somewhat helpful	5 (11.6)	2 (10.0)	3 (13.0)	
Helpful	9 (20.9)	2 (10.0)	7 (30.4)	
Very helpful	29 (67.4)	16 (80.0)	13 (56.5)	
*How helpful was program for learning about PrEP*?				0.03
Somewhat helpful	2 (4.7)	0 (0.0)	2 (8.7)	
Helpful	12 (27.9)	3 (15.0)	9 (39.1)	
Very helpful	29 (67.4)	17 (85.0)	12 (52.2)	
*Quality of the information provided*				0.00
Poor/adequate	0 (0.0)	0 (0.0)	0 (0.0)	
Good	11 (25.6)	0 (0.0)	11 (47.8)	
Excellent	32 (74.4)	20 (100.0)	12 (52.2)	
*Usefulness of the information provided*				0.43
Poor/adequate	1 (2.3)	1 (5.0)	0 (0.0)	
Good	11 (25.6)	3 (15.0)	8 (34.8)	
Excellent	31 (72.1)	16 (80.0)	15 (65.2)	

*Based on non-parametric Mann Whitney U test

### Qualitative results

Most qualitative query evoked responses that were aligned with three HBM constructs: Likelihood of Action, Self-efficacy, and Cues-to-Action. The themes and corresponding quotes that emerged were:

#### Pricey pill (likelihood of action)

Black college women communicated that perceived cost of PrEP was a barrier to initiation.

*“If we were offered [PrEP] for free, and it was something to prevent getting HIV, I wouldn’t have a problem [taking it].”* (Black Female, 19)

*“You’ve got to have money to get those pills. People who have Medicaid, we’re not going to be qualified to get that pill. It’s just for people who got money.”* (Black Female, 21)

*“So, that pill is adding’ on to that****rich****money.”*

#### Pass on the pill PrEParation (likelihood of action)

Black college women agreed that taking a pill would be cumbersome, and their impression of PrEP’s relevance to them was not substantial enough to motivate them beyond the inconvenience of taking another pill.

*“But I feel like, as for me and my friend group, we wouldn’t take it. Because I think, as women, we take so many pills. You would be hesitant [to take another pill] -- you take contraception, you take the morning after pills, and then you have to take this pill [PrEP]. It just seems like a lot… If they could make an injection or something …*” (Black Female, 20)

“*Sometimes the side effects are worse than the medicine itself.”* (Black Female, 19)

#### Rapid remedy vs. accelerating accountability (self-efficacy)

Black college women in this study expressed that PrEP was an effort by the healthcare community to evade having conversations with them about HIV and other sexual/reproductive health prevention solutions, but rather healthcare systems expect them to unwittingly take a pill. In addition, the PrEP pill was perceived by participants as an avoidance of personal accountability for your actions.

*“So … it’s like, we’re just going to give you another pill so you can spend more money to take this pill versus knowing the entire prevention piece. If we can promote everything else, we can promote HIV prevention for Black women … You see breast cancer awareness all year round when they’re collecting money for breast cancer. Why don’t we see that for HIV? And why aren’t we a part of the HIV prevention conversation?”* (Black Female, 21)

*“Just my personal opinion, we should educate more. I use myself as an example. Say I’m going to take this pill. I’m taking this pill to prevent HIV, but I’m not taking this pill to prevent gonorrhea, chlamydia, all the rest of the STDs. We’re putting a Band-Aid over HIV, but we’ve got all the other things [like STDs] out here.”* (Black Female, 20)

*“I feel [PrEP] is a Band-aid, it’s almost like you are promoting a certain lifestyle.”* (Black Female, 22)

*“It’s either right or wrong, but there’s no wrong anymore. Everything is right, it’s okay to do everything, and here’s something to help you with it. You want to prevent HIV? Take PrEP.”* (Black Female, 21)

#### Marketed for men (cues-to-action)

Although some participants were aware of PrEP or saw advertisements about PrEP, they overwhelmingly expressed the same sentiment quoted below. PrEP is marketed to gay men.

*“Yeah, it had gay males in the commercial, but it also showed a woman and it actually shows her with a man. It showed two men, it showed lesbian women, that’s why I thought it was for everybody. But she’s right, it did show more favoritism to gay men.”* (Black Female, 19)

## Discussion

Our PrEP education modules constitute a reliable and cultural−/age-appropriate approach to PrEP information dissemination that celebrates the unique experiences of Black female college students. Students in this study were primarily classified as sophomores and juniors who identified overwhelming as heterosexual and single having 1 to 4 sexual partners in their lifetime. The majority of participants reported that they were HIV negative (79.1%); yet over 50% reported never being tested for HIV.

Prior to intervention engagement, most participants had not heard of PrEP and were not sure or unlikely to take PrEP. Post intervention, most participants reported being likely to take PrEP. PrEP knowledge and PrEP intentions in this sample improved; yet, like in other studies participants’ felt that their self-efficacy to initiate or adhere to PrEP would be undermined by perceived barriers like PrEP cost and PrEP formulation (e.g. pill) [8, 9, 26, 27].

In addition, we determined that our education intervention was feasible and acceptable in both modalities (online and in-person), with the in-person vehicle for intervention delivery more positively received relative to online delivery.

### Feasibility and acceptability

Interpersonal educational engagement is often preferred in Black college women, a sub-population who are perceived to be technological connoisseurs of information (Author publication, 2017; Author publication, 2013b). There were significant differences (*p* < .05) post PrEP education intervention by modality for 1) How helpful was the program for learning about PrEP?, where in-person scores were reported as very helpful (85%) and helpful (15%). The online intervention was also well received, yet there was more variation in the strength of positive responses (very helpful (52%) and helpful (39.1%). 2) There were also significant differences in participant perceptions of the quality of information provided, by modality. Participants in the in-person program unanimously reported that the information was excellent. Although significant, the difference in quality and helpfulness of the PrEP education intervention by delivery modality were indistinct, when considering the descriptors selected by participants. We assert that the online option is a viable alternative to in-person sessions; participants responded to the online module with all favorable descriptors. Based on our previous studies with this population, we are aware that Black college women prefer interpersonal interactions to learn about HIV [38]. Due to budgetary constraints, the online module consisted of still images and required participants to read text. We recommend that future studies consider incorporating animation, audio, and interactive features such as voiced-over dialogue that mimic in-person interactions.

### HIV risk perception in black college women

Considering the discrepancy in the number of participants who had never been tested for HIV and the well documented low HIV risk perception in Black women, internalized HIV stigma (e.g., status, testing) and intentions for HIV/STI testing should be explored further. Intersectionality of Black college women’s social- and behavioral- factors for health promotion and overt/covert messaging of what communities should be concerned about the HIV continuum, made aware of PrEP services, or prescribed/take PrEP is another topic for future research. Especially given the fact that, to date, there have been no PrEP efficacy clinical trials with Black women in the US. According to women in this study, Black women of African ancestry in the US see distinct differences between themselves and African women and expect an appreciable level of research attention and discussions that involve them prior to making decisions about PrEP.

*PrEP knowledge*. Because most of the participants were unaware of PrEP prior to participating in our study, the culturally- and gender- appropriate PrEP education intervention was their initial introduction to a biomedical HIV prevention option. The PrEP module was comprised of content that considered aggregate characteristics of Black college women and their HIV prevention needs (Author publication, 2017; Author publication, 2016), thus we infer that participants were receptive to the content. Superficial, dismissive, and/ or vague PrEP prevention messages can foster misperceptions about who could benefit from PrEP. For instance, participants indicated that their impression of PrEP was that it was a medication for gay men; however, recent advertisements made efforts to include women who projected a comparable identity (e.g. cis-gender, Black ethnicity/culture). The delayed PrEP promotion for Black women was perceived by participants as suspicious and described as though biomedical prevention for Black women is often considered an ‘after thought’ which can, arguably, create feelings of anger, resentment and distrust toward PrEP systems of care. Although knowledge alone does not detour HIV risk behavior, ensuring that populations most susceptible to HIV acquisition are privy to all available HIV prevention options should be a public health priority.

#### PrEP intentions

Parallel to the outcomes of other studies [8, 9], after PrEP education, participants will consider PrEP use. Provoking PrEP intentions in Black college women, was the prospect of an alternative medication formulation (e.g. injectable), rather than a pill [43]. Our study affirms that Black college women, regardless of sociodemographic factors, will consider PrEP use if they are informed by trusted sources.

#### PrEP efficacy

Black women want to partner in HIV prevention efforts that target them. Post exposure to the PrEP education module, participants possessed PrEP knowledge and intentions; however, their PrEP efficacy was dwarfed by well-established barriers to use.

Considering scalability and employing the modules in Student Health Services (SHS) across the nation, we will over-emphasize the existing content that dispels personal-level PrEP use barriers (e.g. knowledge, funding) and enhance the perceived advantages (e.g. female controlled method, empowerment) [43, 44].

#### Limitations

Our study is not without limitations, the first being that the surveys used to examine PrEP knowledge, intention, and efficacy amongst study participants were not validated, thus posing a threat to internal validity. The second limitation is that the Black women in our study were recruited from college campuses in the South, thus our results may not be applicable to Black college students in other geographic locations. Being that our study took place at a HBCU, our findings also cannot be generalized to Black women attending predominantly white institutions or non-HBCUs.

## Conclusions

This culturally relevant and age appropriate PrEP education intervention was feasible and acceptable to Black college women. Given the dearth of research assessing HIV prevention among Black college women, coupled with Black college women’s high HIV risk, there is a need for the development of HIV prevention interventions that are tailored towards this population. PrEP education that is culturally and contextually relevant can fill in gaps in knowledge and promote PrEP uptake among Black women who otherwise may have been hesitant in taking PrEP due to lack of information and misinformation. Ultimately, we anticipate that the PrEP education modules will be a blueprint for developing education tools for other at-risk unengaged populations. Factors of HBCU learning environment that we want to emulate/parallel: promotion of self-identity; [faculty/staff] recognition of social determinants of health, cultural references, learning styles, future perspective, cross-generational/cross-HIV status mentorship, fostering pride and creating an atmosphere of self-reflection, circumspection, and accountability while minimizing the appearance of being a negative representation of the institution and one’s community. Future research should also consider PrEP educational interventions for Black college women attending predominantly white institutions given that the microenvironment that exists on the campuses of predominantly white institutions, coupled with Black women’s experiences on such campuses, may play a role in their knowledge, perspectives, and attitudes towards PrEP initiation and HIV prevention.

## Supplementary information

**Additional file 1.** Focus group guide. This additional file provides detailed information on the guide that was used to conduct focus groups with study participants.

## Data Availability

The datasets during and/or analyzed during the current study are available from the corresponding author on reasonable request.

## Abbreviations

PrEP: Pre-exposure prophylaxis
MSM: Men who have sex with men
HBCU: Historically Black College/University
STI: Sexually transmitted infection
HBM: Health Belief Model
