# Supplementary material for: The pre-exposure prophylaxis (PrEP) consciousness of black college women and the perceived hesitancy of public health institutions to curtail HIV in black women
Source: BMC Public Health. 2020 Jul 28;20:1172. doi: 10.1186/s12889-020-09248-6 (PMC7385954; doi:10.1186/s12889-020-09248-6)
Supplement: Supplementary file 1 — Additional file 1. Focus group guide. This additional file provides detailed information on the guide that was used to conduct focus groups with study participants. [file 12889_2020_9248_MOESM1_ESM.docx]

**Focus Group Guide**

**Introduction:**

Thank you for coming to talk with us today. We appreciate you taking time from your day to help us learn more about keeping young women your age healthy.

My name is ____ and this is ____. We will be working together to help the group go smoothly.

This meeting will last around **1 hour and 30 minutes**.

*We ask that you please turn off your cell phones so you don’t get interrupted while we’re talking. If you can’t turn off your phone for safety reasons, please put it on vibrate only.*

**Group Guidelines:**

Before we start, we need to **agree** on some guidelines to make sure that everyone feels comfortable.

We’ll be talking about **private** information in this group, and it’s important that you all feel that what you share will not be shared with others. So, please don’t share what’s been said here with people who are not in the group.

It’s important that we show **respect** for each other in the group. We will sometimes disagree with each other and that’s normal. We’ll ask that you listen to others, even if you disagree. We will also want to hear your views. Also, please try not to speak when someone else is talking. We want to hear from everyone in the group: you all have something important to add.

We ask that you be as **open and honest** as you can in the group. We can learn from each other, and everyone has something valuable to share. Also keep your mind open to other points of views. There are no right or wrong answers to these questions.

*You may ask questions. We will try to answer any questions when they are asked, but we may need to wait until the end of group if the answer is complicated or doesn’t have to do with the goals of the group.*

*All group sessions will be digitally recorded.*

*Your privacy and confidentiality will be protected at all times. Only the research team, including the transcribers, which are people who translate the audio tapings into a written document, will listen to the tapes.*

*Participants may choose to use their first name only or an alias (alternative) name. Please do not use last names of yourselves or others (including people in your lives). Do you have questions about the taping?*

**Can we all agree to these guidelines?**

**Preliminary Question:**

Let’s go around the group and introduce ourselves. Please use a first name or alias only.

My name is ____ and I work at ____ as a Nurse. Now I will let (co-moderator) introduce themselves.

As we mentioned earlier, we will be working together to help the group go smoothly. (co-moderator) will also be taking notes so we don’t miss any important information.

**Purpose and Goals:**

This is the main goal for tonight’s group:

1. To learn what all of you think about using a medication that can prevent HIV, commonly referred to as PrEP.

*Some of the things that we’re going to be talking about, like sex, can be embarrassing or difficult to talk about. We understand this, and we will help each other to be more comfortable. One more thing about talking about sex—it is very important for group members to feel that their behavior or choices are not being judged. There is a wide range of sexual behaviors. We're all responsible for making our own choices; in this group, we will also be accepting of others' choices. You have the opportunity to learn a lot from each other - as long as members feel comfortable sharing.*

- What type of information do you think you and/or women like you need to know about sex and reproductive health?
- What information have you discussed about sex with your parents?
- What information have you discussed about sex with your peers?
- What information have you heard about HIV prevention in general?
- Tell me all that you know about PrEP?
- What have you heard about PrEP? Where did you get that information?
- What do you think about PrEP?
- What about PrEP would be a barrier for you taking it?
- What about PrEP would encourage you to take it?
- Are you comfortable discussing PrEP with your providers?

**Transition: Recruitment & Retention**

*We really appreciate all the information you have given us. Thank you very much. We have one final area that we would like to talk about. If we were to do a study with Black women focused on sex practices and preventing unintended pregnancies using technology as well as teaching them information about preventing STIs/HIV (Recruitment/Retention):*

- What would be good ways to tell young women about the study?
- What would be a good logos or labels? —Any current logos or labels that you can compare to (What would need to be included in the logo to get your attention?) (Have some options that can be selected)
- What should we call it?
- What should we tell young women to get them interested?
- What do we need to know about what makes it hard participate in a study? Are there days that are not good? Are there times that are not good?

**Final Questions:**

- Is there anything that we didn’t talk about today that you think it would be important for use to know? Did we miss anything?
- If you would like the results of the study, let myself or (RA name here) know, when you get your incentive.

**Closing:**

Thank you very much for coming and talking with us today. We are glad that you were willing to share your thoughts and information with us to help other young women. We will be having other opportunities to participate in this study in the future. If you know of young Black women between the ages of 18 and 29 who might be interested in participating, please tell her about it.
